# A Unique Case of an Expanded Polytetrafluoroethylene Graft Rupture 14 Years after Abdominal Aortic Aneurysm Open Repair Managed with Placement of a Thoracic Endograft

**DOI:** 10.1055/s-0041-1736652

**Published:** 2021-12-28

**Authors:** Loukia Alexopoulou-Prounia, Stavros K. Kakkos, Chrysanthi P. Papageorgopoulou, Konstantinos Katsanos, Peter Zampakis, Polyzois Tsantrizos, Ioannis Ntouvas

**Affiliations:** 1 Department of Vascular Surgery, University of Patras Medical School, Patras, Greece; 2 Department of Radiology, University of Patras Medical School, Patras, Greece

**Keywords:** abdominal aortic aneurysm, endovascular repair, graft rupture, iliolumbar embolization

## Abstract

We report a unique case of expanded polytetrafluoroethylene (ePTFE) tube graft rupture that occurred 14 years after abdominal aortic aneurysm (AAA) repair. Endovascular repair with a thoracic endograft was performed. Postoperatively, an increase in the size of the existing hematoma with active extravasation occurred and was managed with iliolumbar artery embolization. Τo the best of our knowledge, rupture of ePTFE graft used for AAA repair has not been reported in the literature.

## Introduction

Among the various prosthetic graft types used for abdominal aortic aneurysm (AAA) repair, polyethylene terephthalate grafts, marketed as Dacron, are commonly used. During the last three decades, expanded polytetrafluoroethylene (ePTFE) grafts have been increasingly used for this purpose. Although cases of ePTFE graft rupture have been reported when used for axillofemoral bypass grafting, 1 2 to date no case of rupture of an ePTFE graft used for AAA repair has been reported in the literature.

## Case Presentation

**Fig. 1:**
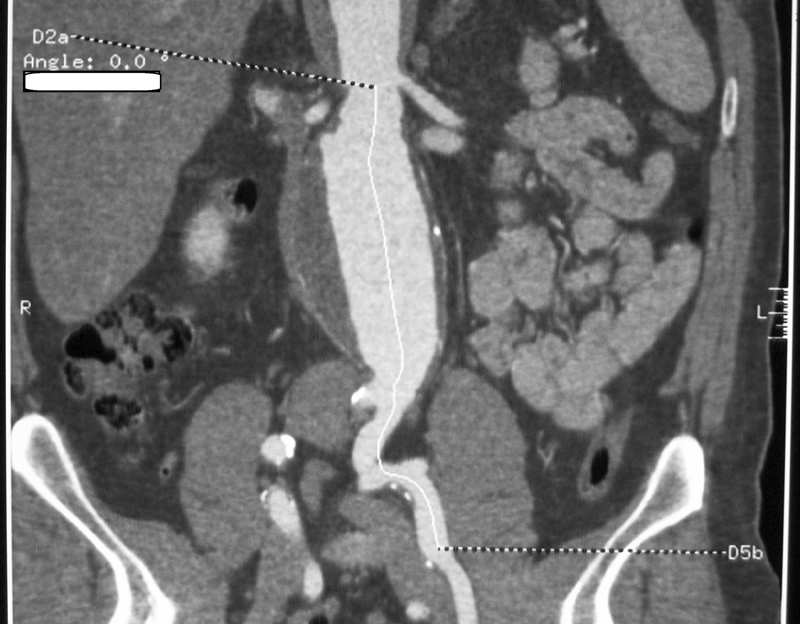
Computed tomography angiography indicates the 1 cm in length reverse taper infrarenal neck of the abdominal aortic aneurysm. No excessive calcium was shown.

A 71-year-old male presented to the emergency department with acute abdominal pain located in the right upper quadrant, nausea, and dizziness. Past medical history included hypertension, dyslipidemia, noninsulin–dependent diabetes mellitus, atrial fibrillation, hyperthyroidism, benign prostatic hyperplasia, left inguinal hernia, and pneumonia. Past surgical history included vocal cord polypectomy and an uneventful elective open repair of an asymptomatic 6.3-cm AAA 14 years ago. On preoperative computed tomography angiography (CTA), the AAA had a 1 cm in length reverse taper infrarenal neck, justifying open repair ( Fig. 1 ) in addition to his young age (57 years). Repair was accomplished with an 18-mm tube ePTFE graft (GORE-TEX stretch vascular graft, W. L Gore & Associates, Flagstaff, Arizona) without complications. There were no known allergies. Social history included smoking (102 pack-years) and alcohol consumption until 14 years ago. Medications included atorvastatin 40-mg once a day, propafenone 150-mg three times a day, metoprolol 12.5-mg twice a day, dabigatran 150-mg twice a day, irbesartan/hydrochlorothiazide 150/12.5-mg once a day, thiamazole 15-mg twice a day, alfuzosin 10-mg once a day, metformin 1,000-mg once a day, and omeprazole 20-mg once a day.

On physical examination, blood pressure was 136/86 mm Hg, pulse rate was 104/min, and temperature was 36°C. A midline incision scar from his previous open AAA repair, as well as obesity, were noted, with no palpable masses. Peripheral pulses were normal.

**Fig. 2:**
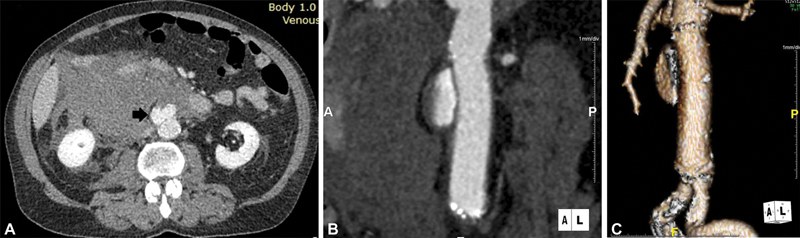
( **A** ) Computed tomography angiography (CTA) of the abdominal aorta showing extravasation of contrast due to graft rupture (arrow) and formation of a large retroperitoneal hematoma. ( **B** ) CTA of the abdominal aorta indicates extravasation of contrast and formation of a 3-cm saccular pseudoaneurysm, 2 cm below the origins of the renal arteries. ( **C** ) Three-dimensional reconstruction CTA of the abdominal aorta and iliac arteries, indicating the exact level of graft disruption.

On laboratory testing, hematocrit was 34.4%; corresponding to an absolute drop of 10% compared with patient's baseline hematocrit level (44.4%), white cell count was 8,590/mm ^3^ , C-reactive protein was 0.23 mg/dL, urea was 73 mg/dL, and creatinine was 1.5 mg/dL. On CTA, the tube graft had a diameter of 2.1 cm, and contrast extravasation was demonstrated from the abdominal aorta at the level of the graft. Adjacent to the graft, a large retroperitoneal hematoma was identified ( Fig. 2 ).

**Fig. 3:**
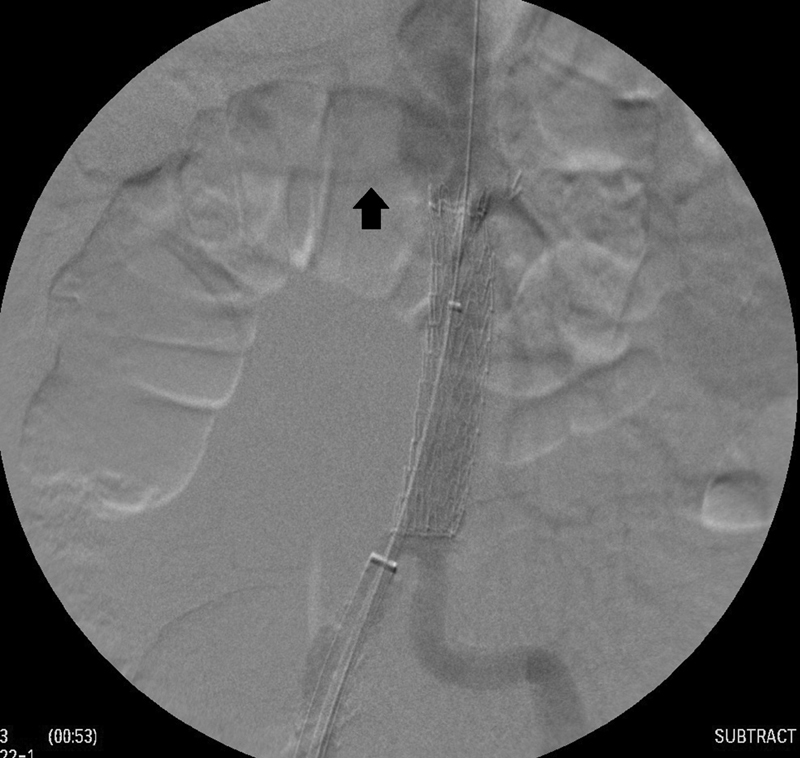
Intraoperative angiogram demonstrates the aortic stent graft appropriately deployed below the renal arteries (arrow showing right renal artery) to the level of the aortic bifurcation.

An endovascular solution was preferred, because of the multiple medical conditions that would potentially complicate open repair. Through a longitudinal groin incision, access to the femoral artery was achieved, and a PTFE-coated standard J-tip guidewire was advanced under fluoroscopic guidance to the thoracic aorta, followed by insertion of an introducer sheath and a marked pig-tail catheter. An abdominal aortography was performed to locate the renal arteries and confirm the length of the infrarenal aortic graft to be covered. We proceeded with exchange of the pig-tail catheter to a vertebral catheter, over a J-wire that was subsequently exchanged to an extra stiff C-tip steerable guidewire. The introducer sheath was exchanged and a 26 mm × 26 mm × 10 cm stent graft (Gore Tag Conformable Thoracic Stent graft, W. L Gore & Associates, Flagstaff, AZ) was advanced and deployed below the renal arteries to the level of the aortic bifurcation ( Fig. 3 ). No endoleak was identified in the final angiography, and the procedure concluded with arteriotomy and wound closure. Fluoroscopy time was 5 minutes and 11 seconds and dose-area product (DAP) was 0.708 Gy cm ^2^ .

**Fig. 4:**
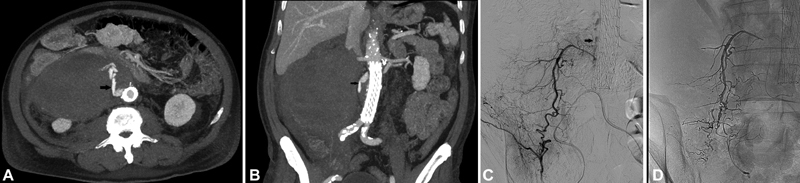
( **Α** and **B** ) Axial and coronal delayed computed tomography images show right-sided active extravasation (arrow) and extensive hematoma. ( **C** and **D** ) Superselective angiography identified the right iliolumbar artery feeding the site of extravasation (arrow), which was successfully embolized with liquid Onyx (microcoils at the top right side were additionally placed inside a higher level lumbar branch that was originally presumed to be a feeder as well).

**Fig. 5:**
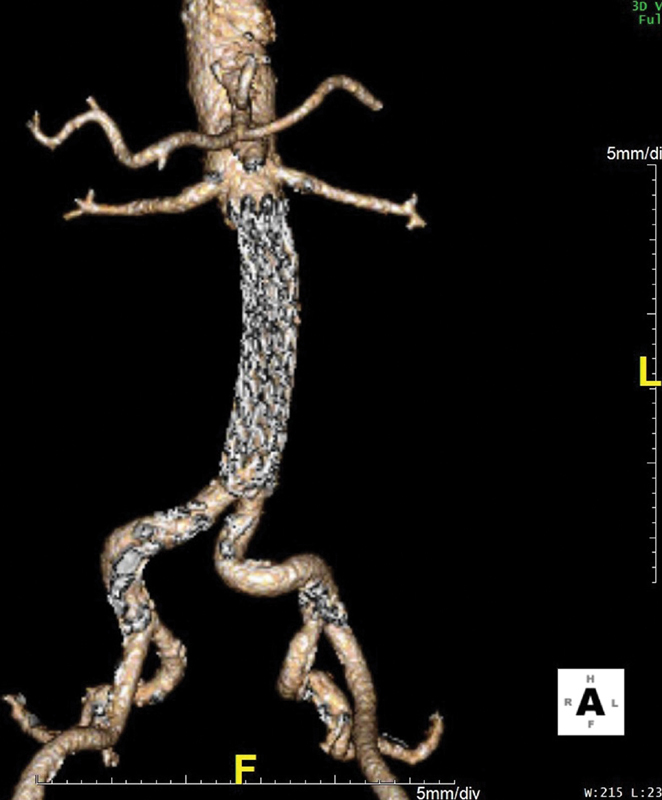
Computed tomography angiography at 37-day follow-up revealed no active extravasation or endoleak. The hematoma was reduced by 2 cm compared with the previous computed tomography.

Postoperatively, the patient was hemodynamically stable and received thromboprophylaxis with 40-mg enoxaparin twice a day. On the third postoperative day, the patient experienced pain in the right abdomen, with a drop of hematocrit from 29 to 27.2% and hemoglobin levels from 9.5 to 8.6 g/dL within less than 2 hours, without hemodynamic compromise. He was transfused with 2 units of red blood cells (RBCs). A CTA revealed an increase in the size of the existing hematoma and active bleeding supplied by the right iliolumbar artery ( Fig. 4A, B ). This was successfully managed with coil embolization of the lumbar branch with a 3.0 mm × 50 mm and 4.0 mm × 120 mm 0.018-inch bare coils (SPIRALES, Balt Spain Medical, Madrid, Spain) and Onyx embolization of the iliolumbar artery (Onyx 18, EV3/Medtronic, Minneapolis, MN; Fig. 4C, D ). The following day, hemoglobin was 7.3 g/dL and hematocrit was 21.8% and he was transfused with another 3 units of RBCs, stabilizing hematocrit to 9.6 g/dL, and hemoglobin to 29.8%. The remaining postoperative course was uneventful, and the patient was discharged home on the 12th postoperative day. He remains in good condition and CTAs on the follow-up at 37th day ( Fig. 5 ) and 10 months revealed no endoleak.

## Discussion

We report a unique case of an ePTFE graft rupture, 14 years after AAA repair that was successfully managed with endovascular repair and iliolumbar embolization.

Graft rupture after AAA repair occurs with Dacron grafts, such as the case of Dacron aortic bifurcation graft failure, 19 years after the implantation (due to graft degradation 3 ). Rupture of a PTFE graft has been reported only when used for axillofemoral bypass repair. The most frequently reported cause of PTFE graft rupture is external force or trauma, with several cases reported due to chronic or acute abrasion and pressure, as well as a rare case of Dacron graft rupture due to friction against a rib. 4 In our patient, no history of trauma or external force was reported.

Graft dilatation of ePTFE tube grafts can be anticipated up to 20% of their initial diameter, after a mean implantation time of 6 years. 1 It has yet to be defined whether graft dilatation grows until reaching a “plateau” or continues to gradually increase until a critical point of expansion, followed by graft rupture. 1 In our case, we did not notice any indication of chronic graft dilation contributing to graft rupture.

Specific surgical maneuvers, such as application of clamps to the graft or the placement of the prosthesis under excessive tension may contribute to its failure. 2 In our case, surgical maneuvers were unlikely to have contributed to the graft rupture, since the location of the rupture was distant from all maneuvers and the clamping area, close to the anastomosis.

Alternatively, a calcified plaque of the AAA sac that is imbricated over the graft may lead to its erosion. In our case, calcification of the AAA sac was not identified.

Our patient underwent a minimally invasive endovascular procedure instead of an open surgery. Therefore, no histologic evaluation could be performed to assess the internal structure of the prosthetic material, as well as the mechanism of the rupture. The diagnosis of the graft rupture was made on CTA with three-dimensional reconstruction, determining the exact level of the graft disruption. We attribute the postoperative bleeding to local tissue avulsion of the right iliolumbar artery branches related to the rapidly expanding retroperitoneal hematoma in conjunction with anticoagulation, although given at intermediate doses. This was managed with coil embolization which has been described also for the treatment of a spontaneous left renal subcapsular hematoma, following complex percutaneous coronary intervention. 5

In conclusion, we present a unique case of ePTFE graft rupture used for open AAA repair, 14 years after its initial implantation, successfully managed with endovascular repair.
